# What is the level of work and societal participation in patients with pelvic ring injuries? A two-year prospective cohort study

**DOI:** 10.1177/02692155251333535

**Published:** 2025-04-23

**Authors:** Camryn C Therrien, Kaj ten Duis, Hester Banierink, NM Trouwborst, Jean-Paul PM de Vries, Frank FA IJpma, Inge HF Reininga

**Affiliations:** 1 Department of Trauma Surgery, University of Groningen, University Medical Center Groningen, Groningen, The Netherlands; 2 Department of Surgery, University of Groningen, 10173 University Medical Center Groningen, Groningen, The Netherlands

**Keywords:** participation (WHO ICF), work, social and leisure activities, pelvis

## Abstract

**Objective:**

To provide insight into the impact of pelvic ring injuries on patients’ work and school activities and participation in society.

**Design:**

Prospective-cohort study.

**Setting:**

A level-1 trauma center in the Netherlands.

**Participants:**

195 patients with a pelvic ring injury.

**Main measures:**

The work or school activities and participation in society domains of the World Health Organization Disability Assessment Score II (WHO-DAS II) were administered at admission (pre-injury score), 3 months, 6 months, 1 year and 2 years following the injury.

**Results:**

Before the injury, the median scores were 80 for both work or school activities and participation in society. The scores 3 months after the injury were 40 and 60, respectively, but both improved to 75 after 2 years. The percentage of non-recovered patients decreased over time, from 45% to 35% for work or school activities and from 34% to 18% for participation in society between 6 months and 2 years. At work or school, patients struggled to complete daily tasks and important activities as efficiently and effectively as needed. Regarding participation in society, patients struggled with the amount of time spent managing their injuries, joining community activities, and doing things for relaxation. No relationships between patients or injury characteristics and recovery were identified one year following the injury.

**Conclusions:**

Pelvic ring injuries greatly impact patients’ work or school activities and participation in society. However, these domains greatly improve within the first two years, with many individuals regaining their pre-injury capabilities. Still, some continue to experience long-term difficulties in participation.

## Introduction

The pelvic ring is composed of two innominate bones and the sacrum, held together by a complex network of ligaments.^1^ Disruptions to the pelvic ring are typically caused by high-energy traumas, such as motor vehicle accidents, in a younger patient population and low-energy traumas, such as falls, in an older patient population.^2^ Pelvic ring injuries have an estimated annual incidence of 14–37 per 100,000 inhabitants.^3^ These injuries often result in a period of immobilization and discomfort, with some patients experiencing long-term physical impairments and decreased quality of life.^4–9^

In order to gain insight into the patient's health and health problems, the World Health Organization's International Classification of Functioning, Disability and Health (ICF) framework is often used.^10–12^ The ICF model emphasizes a comprehensive view of health, considering not just medical conditions but also functional abilities and participation in daily life. This is crucial for pelvic ring injuries, as they can significantly affect mobility and quality of life. The abundance of literature on participation in other patient groups, such as traumatic spinal cord or brain injury or stroke, highlights the importance of this focus in the rehabilitation process.^13–17^ However, while there is ample literature on the impact of pelvic ring injuries on the patient's body functions, structure and the patient's level of activity,^4–9^ research on the impact on work and social participation is lacking.

Therefore, a six-year-long prospective study was conducted at a single level-1 trauma and referral center for the treatment of pelvic ring injuries. Our research questions were: (1) What is the course of recovery regarding work or school activities and participation in society within the first two years after a pelvic ring injury? (2) What did the patients struggle with the most at three months, six months, one year and two years following the injury? (3) Are there predictors for a lower level of recovery regarding work or school activities or participation in society after one year?

## Methods

This prospective longitudinal cohort study was performed over six years (2017–2023) on patients treated for pelvic ring injuries. This took place at a level-1 trauma and referral center in the Netherlands. The local Medical Ethical Review Board reviewed the methods employed and waived further need for approval (METc 2017/543).

**Figure 1.**
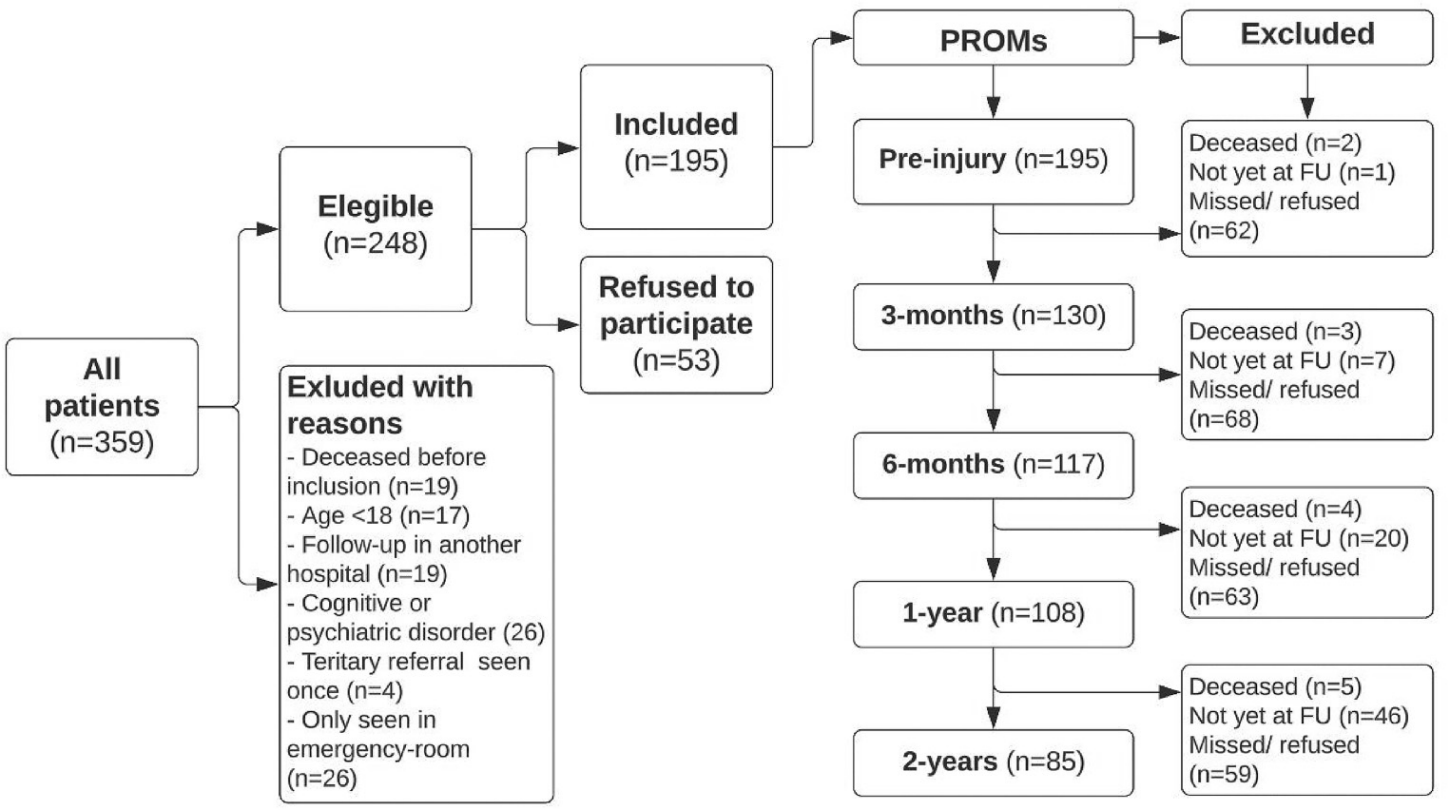
The patient inclusion process for this study is depicted in this flowchart.

All patients above 18 years of age, without cognitive disorders and who could read and understand Dutch were informed about the study and asked to participate. Further exclusion criteria can be found in Figure 1.

Data on the patients’ characteristics were prospectively collected from the patients’ electronic records and directly entered into the database upon clinical presentation. These include information regarding the patient demographics, injury, treatment, and mortality, such as gender, age, the occurrence of high energy trauma (HET) (defined as a fall from a height of two to three times the height of the patient or an impact of 20 km/h or greater), an injury severity score (ISS) of 16 or greater, the presence of an isolated pelvic ring injury (without additional fractures), associated lower extremity injuries, emergency laparotomy, embolization, use of an external fixator, surgical treatment, and whether operative fixations were placed anteriorly, posteriorly, or both. Radiographs were also retrieved from the electronic records.

Two trauma surgeons with ample experience in pelvic ring injury surgery assessed the radiographic images (plain anteroposterior, inlet and outlet radiographs and CT scans) of all the patients. Injuries were classified according to the AO Foundation/Orthopaedic Trauma Association classification as type A, B or C.^18^ Type A injuries are stable, type B are partially stable, and type C are completely unstable injuries.^18^

### Patient-reported outcomes

The World Health Organization Disability Assessment Score II (WHO-DAS II) questionnaire was used to investigate the participation component of the ICF framework in terms of work or school activities and participation in society. The WHO-DAS II is a valid questionnaire for orthopaedic and trauma patients that is directly linked to the ICF framework, covering six disability domains.^19–22^ In this study, the domains work or school activities (4 items) and participation in society (8 items) were used to assess the level and difficulties regarding work or school activities and participation in society, respectively. The WHO-DAS II uses a 5-point Likert scale for each item, ranging from “none” (1 point) to “extreme” (5 points). Sum scores for work or school activities and participation in society were converted to a 100-point score, with a higher score representing a better outcome.

The two domains of the WHO-DAS-II questionnaire were administered digitally at five time points post-injury: during hospital admission, 3 months, 6 months, 1 year, and 2 years after the injury. During admission, the so-called pre-injury status on work or school activities and participation in society was administered to assess the baseline level of participation of the participants. The WHO-DAS-II was collected using a secure online system, RoQua, which was linked to the electronic patient files. This system provides a personal code which is linked to a secure website and allows patients to complete the digital WHO-DAS-II at home or during their follow-up visits at the pelvic outpatient clinic.

### Statistical analysis

The statistical analysis was performed using SPSS (version 28, IBM Corp). Descriptive statistics were performed to present patient and injury characteristics and the scores at each follow-up moment. Means and standard deviations were used with normally distributed data and the median and interquartile range (IQR) with non-normally distributed data.

The course and level of recovery (expressed as % of the pre-injury WHO-DAS-II score), number of patients considered fully recovered, as well as the difficulties experienced in work or school activities and participation in society, were presented for all follow-up moments. At every follow-up moment, each patient's score was represented as a percentage relative to the pre-injury score to indicate the level of recovery. Additionally, patients were classified as “recovered” if the difference between their score at that follow-up moment and at pre-injury was less than 15 points.

The difficulties are identified as the items within the work or school activities and participation in society domains of the WHO-DAS-II where patients often report having extreme difficulties (defined as a 4 or 5 on the 5-point Likert scale). The items are presented in a table, ranked 1^st^ to 4^th^ most difficult by the percentage of patients who perceived that item as severely difficult. The items have been summarized in the table, however, the complete, official English translation of the items from the questionnaire can be found in the supplementary data.

To identify potential predictors of recovery of work or school activities and participation in society, the first step was to perform univariate logistic regression analyses with the following patient and injury characteristics: age, gender, type of injury (type A, B or C), surgical treatment, isolated pelvic injury, HET, and ISS ≥** **16. Predictors with a p-value <0.20 were then included in a multivariate logistic regression analysis (backward selection).

## Results

### Patients

Throughout the six years of the study, a total of 359 patients were treated for pelvic ring injury. Two-hundred and forty-eight of these patients (69%) were eligible to participate, wherefrom 53 (21%) did not wish to participate. The remainder of the patients was excluded due to reasons stated in Figure 1. There were ultimately 195 patients who agreed to participate and who completed one or more follow-up questionnaires. Patient and injury characteristics can be found in Table 1.

**Table 1.** Patient and injury characteristics of patients with pelvic ring injuries.

Patient and injury characteristics	Patients included (*n* = 195)
Women, *n* (%)	105 (54)
Age at the time of injury, mean (SD)	58 (18)
High energy trauma, *n* (%)	122 (63)
ISS ≥ 16, *n* (%)	41 (21)
Injury type, *n* (%)*	
Type A	59 (30)
Type B	109 (56)
Type C	27 (14)
Isolated pelvic ring injury, *n* (%)	91 (47)
Associated lower extremity injuries, *n* (%)	26 (13)
Emergency laparotomy, *n* (%)	2 (1)
Embolization, *n* (%)	4 (2)
External fixator, *n* (%)	7 (4)
Operative treatment, *n* (%)	55 (28)
Fixation location, *n* (%)	
Anterior fixation	21 (11)
Posterior fixation	14 (7)
Anterior–posterior fixation	20 (10)
Deceased <1 year, *n* (%)	9 (1)
Deceased <30 days, *n* (%)	0

* Injuries were classified using the AO Foundation/Orthopaedic Trauma Association classification system: type A injuries are stable, type B are partially stable, and type C are completely unstable injuries.^18^

### Patient-reported work or school activities and participation in society

**Figure 2.**
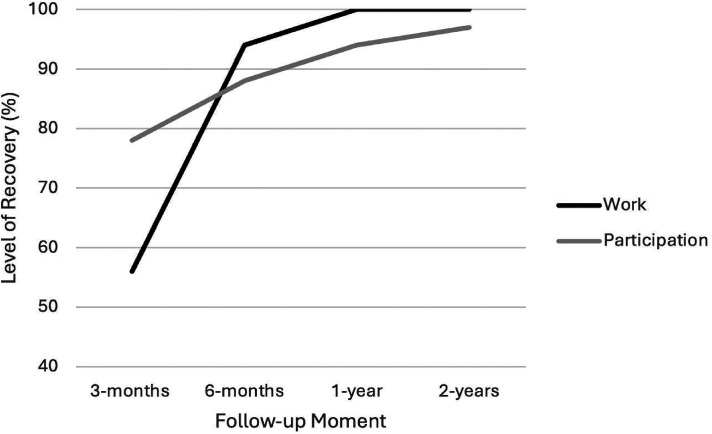
Median level of recovery represented as the score at that follow-up moment expressed as a percentage of the mean pre-injury WHO-DAS-II score for the work or school activities and participation in society domains at the different follow-up moments.

**Figure 3.**
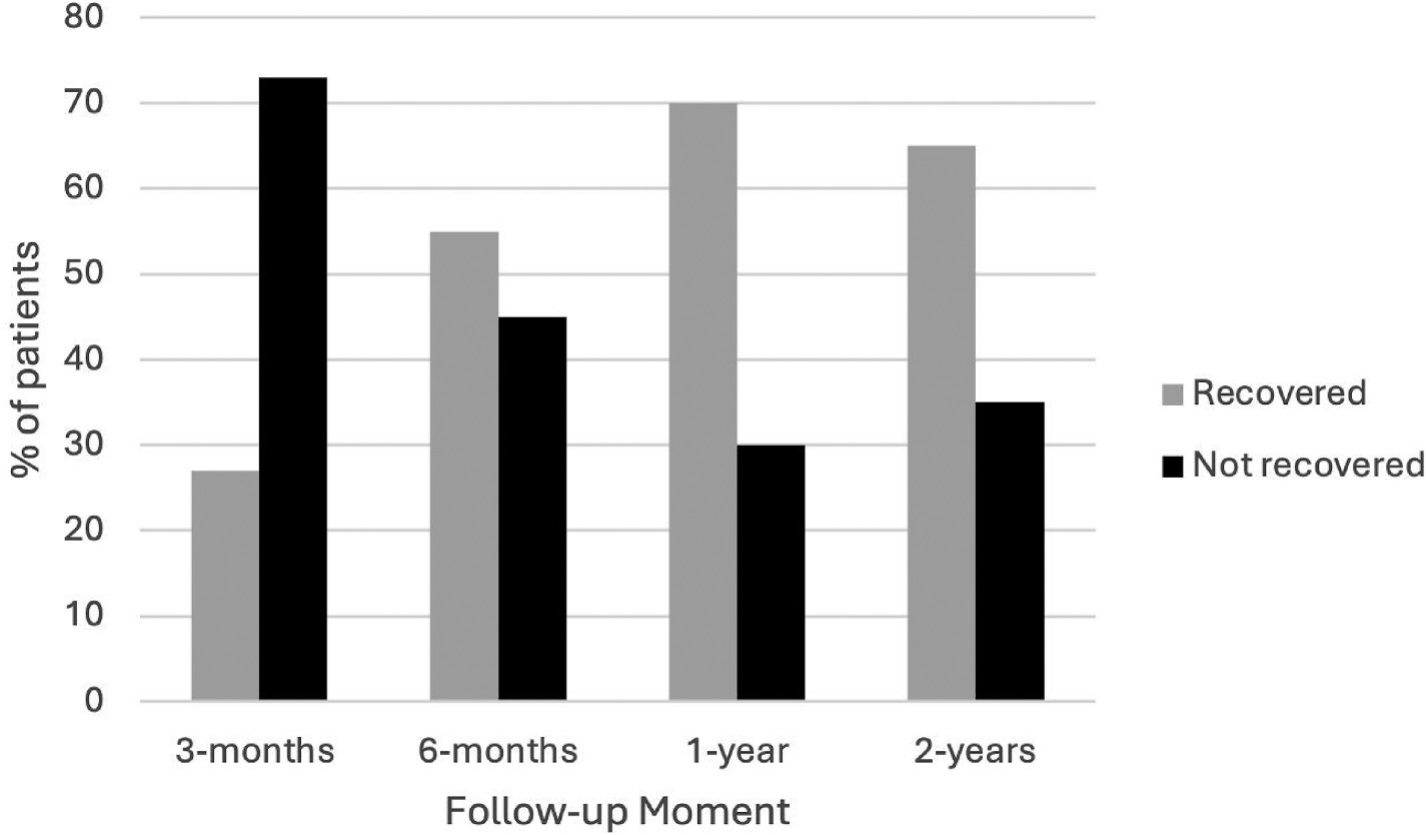
Percentage of recovered patients in the work or school activities domain. Recovered patients have a score at that follow-up moment that is within 15 points of the pre-injury score.

**Figure 4.**
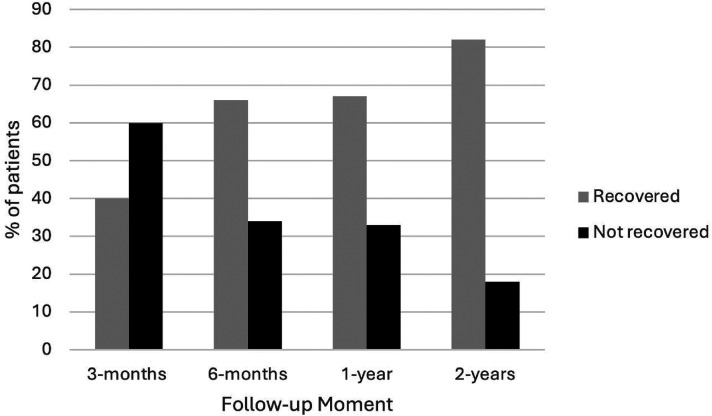
Percentage of recovered patients in the participation in society domain. Recovered patients have a score at that follow-up moment that is within 15 points of the pre-injury score.

The work or school activities and participation in society scores are presented in Table 2, along with the median level of recovery and the number of patients fully recovered at that follow-up moment. The median level of recovery is shown in Figure 2. The median for work or school activities reached 100% by the one-year follow-up, however, the one- and two-year follow-up results for participation in society were 94% and 97%, respectively. The number of patients who recovered or not at each follow-up is graphically presented in Figure 3 for work or school activities and in Figure 4 for participation in society. At the one-year and two-year follow-up moments, respectively, 26 (70%) and 20 (65%) patients recovered with work or school activities, and 51 (67%) and 46 (82%) patients were recovered with participation in society.

**Table 2.** WHO-DAS II score and level of recovery at all follow-up moments in patients with pelvic ring injuries.

	Work or school activities			Participation in society		
Follow-up moment	Score, median (IQR)	Level of recovery*, median (IQR)	Number of patients fully recovered *N* (%)	Score, median (IQR)	Level of recovery*, median (IQR)	Number of patients fully recovered *N* (%)
Pre-injury	80 (0)	—	—	80 (10)	—	—
3-months	40 (29)	56 (63)	11 (27)	60 (25)	78 (27)	39 (40)
6-months	70 (30)	94 (50)	28 (55)	68 (20)	88 (25)	58 (66)
1-year	75 (20)	100 (25)	26 (70)	69 (20)	94 (82)	51 (67)
2-years	75 (20)	100 (25)	20 (65)	75 (14)	97 (15)	46 (82)

* Level of recovery is the score at that follow-up moment expressed as a percentage of the pre-injury score.

### Difficulties experienced with work or school activities

A severity ranking, along with the percentage of patients who experienced severe difficulties with that item of the work or school activities domain at each follow-up moment, can be seen in Table 3. At three out of the four follow-up moments, the highest ranked difficulty was the item regarding difficulty with completing day-to-day work. Other items that were high on the severity ranking were the patient's ability to do their important tasks well and also getting work done as quickly as needed.

**Table 3.** Difficulties experienced with work or school activities at 3 and 6 months, 1 year, and 2 years of follow-up after a pelvic ring injury.

Severity rank	3-months	%*	6-months	%*	1-year	%*	2-years	%*
1	Difficulty with day-to-day work	35	Doing important tasks well	21	Difficulty with day-to-day work	4	Difficulty with day-to-day work	12
2	Doing important tasks well	33	Getting work done quickly	21	Doing important tasks well	4	Doing important tasks well	10
3	Finishing what has to be done	32	Finishing what has to be done	18	Finishing what has to be done	4	Getting work done quickly	10
4	Getting work done quickly	25	Difficulty with day-to-day work	17	Getting work done quickly	4	Finishing what has to be done	8

* Percentage of patients that experience severe difficulties (severe difficulty is assumed by a score of 4 or 5 on the 5-point scale for each question).

### Difficulties experienced with participation in society

A severity ranking, along with the percentage of patients who experienced severe difficulties with that item of the participation in society domain at each follow-up moment, can be seen in Table 4. At all four follow-up moments, the item that patients struggled with the most was the amount of time they had to spend on their health condition, meaning the time spent managing their injury. Other important items perceived as severely difficult were joining community activities (for example, festivities, religious or other activities) and problems with doing things by themselves for relaxation or pleasure.

**Table 4.** Difficulties experienced with participation in society, 3 and 6 months, 1 year, and 2 years of follow-up after a pelvic ring injury.

Severity rank	3-months	%*	6-months	%*	1-year	%*	2-years	%*
1	Time spent on condition	47	Time spent on condition	29	Time spent on condition	21	Time spent on condition	22
2	Joining community activities	35	Time alone for relaxation or pleasure	16	Joining community activities	12	Joining community activities	11
3	Time alone for relaxation or pleasure	25	Joining community activities	14	Time alone for relaxation or pleasure	12	Barriers in surroundings	11
4	Barriers in surroundings	17	Family problems	10	Barriers in surroundings	10	Time alone for relaxation or pleasure	11

* Percentage of patients that experience severe difficulties (severe difficultsy is assumed by a score of 4 or 5 on the 5-point scale for each question).

### Predictors for no recovery one year following the injury

While performing the univariate logistic regression analyses with the following patient and injury characteristics: age, gender, type of injury (type A, B or C), surgical treatment, isolated pelvic injury, HET, and ISS ≥** **16, no variables had a p-value less than 0.20. Therefore, a multivariate linear regression analysis could not be performed, and no significant predictors were found for recovery one year following the injury.

## Discussion

Patients with pelvic ring injuries experience significant difficulties in work or school activities and social participation, particularly in the early stages of recovery. Compared to the pre-injury scores, both work and social participation were substantially impacted by the traumatic pelvic injury at the three-month follow-up moment. Nonetheless, both domains gradually improved over the first two years of the recovery process. Despite the improvement, a substantial proportion of the patients did not recover within two years. At the two-year follow-up moment, 35% of the patients were not recovered at work or school and 18% were not recovered with participation in society.

Regarding work or school activities, patients experienced the most difficulty with completing daily work, doing their important tasks well and getting work done as quickly as needed. This is of importance in the recovery process as patients with less work participation tend to have a worse perception of their illness.^23^ Additionally, motivational processes experienced during work, such as goal selection and pursuit, are crucial factors in health and well-being.^24^ If patients struggle to feel a sense of motivation and accomplishment at work, it can negatively impact their health and recovery. This is the first study that uses the WHO-DAS-II questionnaire to investigate work participation following a pelvic ring injury. Multiple studies have explored the effect of a pelvic ring injury on a patient's return to work,^25–27^ however, this is the only study providing insights into the specific components of work that patients struggle with.

Regarding participation in society, it was concluded that the patients experienced the most difficulties with the amount of time they had to spend managing their injury, joining community activities (for example, festivities, religious or other activities), and problems with doing things by themselves for relaxation or pleasure. It is important to consider the amount of time patients spend on health-related activities, as this has been shown to have a critical burden on patients with chronic health conditions.^28^ Because of the time spent on their condition, patients lack the time and ability to do things for relaxation or pleasure (activating a relaxation response), which has a negative effect on some health problems.^29^ Additionally, the patient's difficulties with participating in community activities are important to consider, as social factors can affect pain and symptom intensity in orthopaedic patients.^30^ Patients should be assessed using a biopsychosocial approach to identify problems in these areas.^31,32^ This is the first study investigating social participation in this patient group. Participation has been thoroughly researched, and the importance has been highlighted in other patient groups, for example, in patients undergoing rehabilitation for a spinal cord injury, brain injury, or stroke.^13–17^ However, attention to this topic is lacking in patients with pelvic ring injuries. Evidence shows that patients with a higher level of social participation also experience a higher level of health and well-being.^33–35^ Given the role that social participation plays in both the ICF framework and the impact it can have on well-being, there should be more emphasis on this in future research for patients with pelvic ring injuries.

No significant predictors of recovery in terms of work or school activities and participation in society one year following the injury could be identified. These results are supported by literature stating that there are many factors, beyond patient and injury characteristics, that influence participation in adults with a physical disability.^36^ Those being: physical environment factors, social factors, symptoms, economic factors, policy factors, body structure and functions, mental and emotional state, and temporal factors.^36^ Given the complexity of the predictors for participation, it may not be possible to predict the patient's level of participation based on information such as the age or gender of the patient or the type or severity of the injury. This, along with other literature,^32^ further describes the necessity of the shift from a biomedical approach to a biopsychosocial approach.

Difficulties in participation should be identified by the surgeon at the 3- and 6-month follow-up moments by means of either a questionnaire or an anamnesis. The most frequently faced difficulties presented in this article should be specifically addressed. Surgeons’ awareness and early detection of difficulties in work and societal participation following pelvic ring injuries provide opportunities to refer these patients early for professional, social or occupational support.

We acknowledge that this study has some degree of response bias, which is inherent to the longitudinal prospective study design, caused by loss to follow-up and nonresponse. Furthermore, we were not able to identify any significant predictors for recovery one year following the injury. With a larger sample size, identifying factors may be possible. Moreover, participation is complex and can be impacted by many factors besides patient and injury characteristics,^36^ which was outside of the scope of this study. Additionally, we were not able to provide an analysis of factors such as ethnicity and socio-economic status to determine how representative the sample is of the Dutch population of patients with pelvic ring fractures. Although this study was conducted in a level-1 trauma and referral center in the north of the Netherlands, which suggests that the sample may be representative of the regional population with pelvic ring injuries, we were unable to assess whether non-responders differed systematically from responders based on ethnicity or socio-economic status. Therefore, the lack of this analysis should be considered a limitation of the study. Furthermore, additional information about the patients’ work, support systems, and living situations could have provided valuable insights into the broader applicability of the findings.

In conclusion, this study demonstrated to what extent pelvic ring injuries impact work or school activities and participation in society during the first two years of recovery. Our results showed that pelvic injuries significantly affect work or school activities and participation in society in the short term, as demonstrated by the decline in scores at the three-month follow-up. However, there is a gradual improvement in these scores over the first two years of recovery. Despite this progress, a substantial proportion of patients did not recover within two years. By the two-year mark, 35% of patients were not recovered in the work or school activities domain, and 18% in the participation in society domain, indicating that while many individuals do regain their pre-injury capabilities, a notable proportion continues to experience long-term impacts. Factors that patients struggled with the most were their daily work, and the amount of time spent on their condition. These long-term impacts on participation should be considered, as participation makes up an important branch of the ICF model and has an impact on a patient's well-being. There were no predictors for recovery identified due to the complexity of factors that influence participation. All patient groups need to be treated with a biopsychosocial approach to optimize their recovery. Awareness of this topic, along with the utilization of questionnaires, could minimize the substantial number of patients who do not recover within two years.

**Clinical messages**

- Pelvic ring injuries greatly impact patients’ work activities and social participation, with a notable proportion experiencing long-term impact.
- The biggest barrier to work or school activities was the patients’ ability to complete daily work.
- The biggest barrier to social participation was the amount of time patients spent managing their injuries.
- Practitioners’ awareness of participation difficulties can help identify patients in need of support and potentially reduce the number of those who do not recover within two years.

## Supplemental Material

sj-tif-1-cre-10.1177_02692155251333535 - Supplemental material for What is the level of work and societal participation in patients with pelvic ring injuries? A two-year prospective cohort study Supplemental material, sj-tif-1-cre-10.1177_02692155251333535 for What is the level of work and societal participation in patients with pelvic ring injuries? A two-year prospective cohort study by Camryn C Therrien, Kaj ten Duis, Hester Banierink, NM Trouwborst, Jean-Paul PM de Vries, Frank FA IJpma and Inge HF Reininga in Clinical Rehabilitation
