# Supplementary figures and images for: What is the level of work and societal participation in patients with pelvic ring injuries? A two-year prospective cohort study
Source: Clin Rehabil. 2025 Apr 23;39(6):808–18. doi: 10.1177/02692155251333535 (PMC12141768; doi:10.1177/02692155251333535)

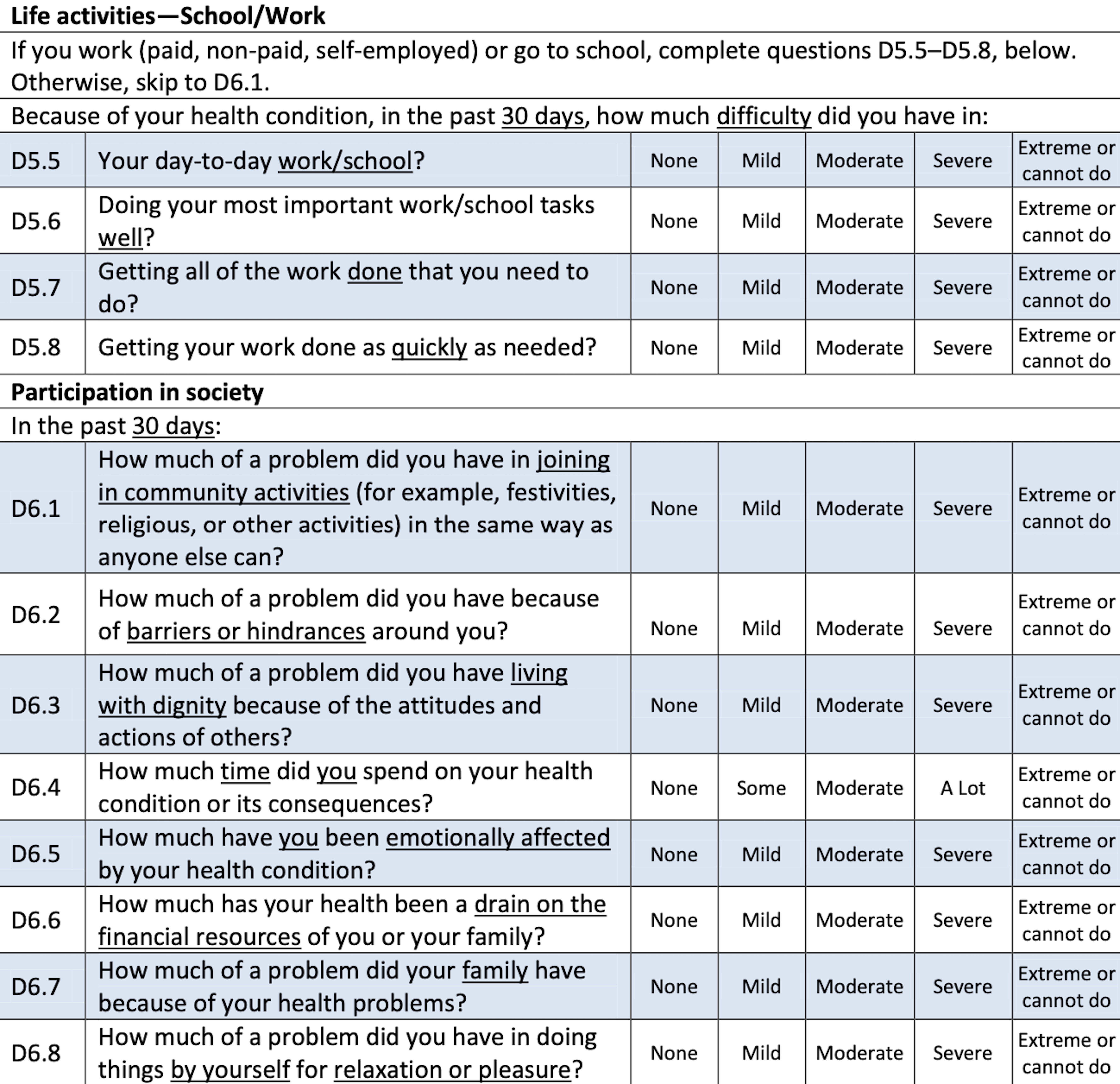

Supplement: sj-tif-1-cre-10.1177_02692155251333535 - Supplemental material for What is the level of work and societal participation in patients with pelvic ring injuries? A two-year prospective cohort study [file sj-tif-1-cre-10.1177_02692155251333535.tif]
